# National Monkeypox Surveillance, Central African Republic, 2001–2021

**DOI:** 10.3201/eid2812.220897

**Published:** 2022-12

**Authors:** Camille Besombes, Festus Mbrenga, Laura Schaeffer, Christian Malaka, Ella Gonofio, Jordi Landier, Ulrich Vickos, Xavier Konamna, Benjamin Selekon, Joella Namsenei Dankpea, Cassandre Von Platen, Franck Gislain Houndjahoue, Daniel Sylver Ouaïmon, Alexandre Hassanin, Nicolas Berthet, Jean-Claude Manuguerra, Antoine Gessain, Arnaud Fontanet, Emmanuel Nakouné-Yandoko

**Affiliations:** Sorbonne Université, Paris, France (C. Besombes);; Institut Pasteur, Paris (C. Besombes, L. Schaeffer, C. Von Platen, N. Berthet, J.-C. Manuguerra, A. Gessain, A. Fontanet);; Institut Pasteur, Bangui, Central African Republic (F. Mbrenga, C. Malaka, E. Gonofio, X. Konamna, B. Selekon, J. Namsenei Dankpea, E. Nakouné Yandoko);; Aix Marseille Université, Marseille, France (J. Landier);; Centre Hospitalier Universitaire, Bangui (F.G. Houndjahoue, D.S. Ouaïmon);; Sorbonne Université, Paris (A. Hassanin);; Institut Pasteur of Shanghai, Shanghai, China (N. Berthet);; Conservatoire National des Arts et Métiers, Paris (A. Fontanet)

**Keywords:** monkeypox, monkeypox virus, Central Africa, Central African Republic, national surveillance, outbreak investigation, zoonoses, emerging infectious diseases, viruses

## Abstract

We analyzed monkeypox disease surveillance in Central African Republic (CAR) during 2001–2021. Surveillance data show 95 suspected outbreaks, 40 of which were confirmed as monkeypox, comprising 99 confirmed and 61 suspected monkeypox cases. After 2018, CAR’s annual rate of confirmed outbreaks increased, and 65% of outbreaks occurred in 2 forested regions bordering the Democratic Republic of the Congo. The median patient age for confirmed cases was 15.5 years. The overall case-fatality ratio was 7.5% (12/160) for confirmed and suspected cases, 9.6% (8/83) for children <16 years of age. Decreasing cross-protective immunity from smallpox vaccination and recent ecologic alterations likely contribute to increased monkeypox outbreaks in Central Africa. High fatality rates associated with monkeypox virus clade I also are a local and international concern. Ongoing investigations of zoonotic sources and environmental changes that increase human exposure could inform practices to prevent monkeypox expansion into local communities and beyond endemic areas.

Monkeypox, caused by monkeypox virus (MPXV), a member of the *Orthopoxvirus* genus, was considered a rare emerging disease before a multinational outbreak was identified in May 2022 (*1*). After global smallpox eradication in 1977, monkeypox became the most concerning human *Orthopoxvirus* infection. Clinical manifestations of monkeypox typically resemble those of smallpox, including a febrile prodrome and subsequent disseminated maculopapular rash, including vesicles and pustules, that occurs in successive stages (*2*). Lymphadenopathy is a prominent feature of monkeypox and usually does not occur for smallpox and chickenpox (*3*). Illness is less severe and death less likely among monkeypox cases than smallpox cases, but monkeypox mortality rates vary and are higher for clade I (formerly the Congo Basin clade) than for clade II (formerly the West African clade) viruses (*2*). Prior smallpox vaccination can confer cross-immunity for monkeypox, but smallpox vaccination programs worldwide ended in the early 1980s (*4*).

In 1970, a human monkeypox case was reported from Basankusu, Equateur Province, Democratic Republic of the Congo (DRC) (*5*). Subsequent sporadic monkeypox cases were reported among human and animal populations from remote areas of Central Africa during the 1970s and 1980s (*6*,*7*). Since 1990, increases in the frequency and scale of epidemics in Africa have been reported for clade I and, to a lesser extent, since 2000 for clade II. Since 2016, confirmed monkeypox cases have been reported in DRC, Central African Republic (CAR), Republic of Congo (hereafter Congo), Nigeria, Sierra Leone, Liberia, and Cameroon (*6*). The true burden, circulation rates, and geographic range of this emerging disease remain unknown because many countries lack systematic routine monkeypox surveillance and affected areas often are remote (*8*,*9*). 

An outbreak of human monkeypox disease occurred outside Africa in 2003, after infected animals from Ghana were imported into the United States (*10*). Since 2018, several self-limited monkeypox outbreaks have been reported among travelers from the United Kingdom, Singapore, Israel, and the United States after travel to Nigeria (*11*) (https://www.cdc.gov/poxvirus/monkeypox/outbreak/us-outbreaks.html). A large worldwide monkeypox outbreak was documented in May 2022 (https://www.who.int/emergencies/situations/monkeypox-oubreak-2022), including interhuman transmission in >85 countries outside Africa and 5 reported monkeypox-related deaths (*12*), highlighting the global public health threat posed by this disease. Primary zoonotic transmission presumably results from at-risk activities, such as hunting or butchering bushmeat or handling animal carcasses (*13*). Rodents, including arboreal rope squirrels (*Funisciurus* spp.) and terrestrial rodents (*Cricetomys* and *Graphiurus* spp.) (*14*), are believed to be the main sources for MPXV introduction into human populations, but the natural virus reservoir remains unknown and molecular sequencing of animal–human pairs has yet to identify the same MPXV strain in both organisms. 

Human-to-human viral transmission seems to occur through direct contact with lesion exudates, bodily fluids, or respiratory droplets; or through indirect contact with environments contaminated by monkeypox patients (*15*). The 2022 outbreak primarily has occurred among men who have sex with men (MSM) and has highlighted the role of direct cutaneous and mucosal contact during sexual intercourse and the potential contribution of sexual transmission (*2*,*16*), but exact modes of transmission remain unclear.

Before the 2022 worldwide outbreak, CAR was fourth among monkeypox-affected countries, after DRC, Nigeria, and Congo (*2*), but epidemiologic data concerning monkeypox in CAR remains scarce (*17*–*24*). We provide a comprehensive analysis of national monkeypox surveillance in CAR during 2001–2021.

## Methods

### National Monkeypox Surveillance and Epidemiologic Outbreak Investigations 

The population of CAR consists mainly subsistence farmers and hunter-gatherers who live in small villages or towns. The Institut Pasteur of Bangui (IPB), the CAR Ministry of Public Health and Population, and the World Health Organization (WHO) established the CAR national monkeypox surveillance system in 2001. Healthcare workers in the field receive regular training on the clinical manifestations of monkeypox and the importance of rapid case identification. These workers send blood, pus, and crust samples from suspected monkeypox cases to IPB, which serves as the national MPXV reference center. After virologic confirmation, IPB deploys an outbreak investigation team to the field to conduct a more thorough investigation of cases and their contacts. The IPB investigation team administers specific case-report questionnaires via paper surveys in the local language, Sango, to collect information about demographic characteristics, socioeconomic status, education, and contact with wildlife or other human cases. A trained practitioner on the IPB team uses sterile techniques to collect swab samples of pus, crusts, or lesions from each suspected case, and 3–5 mL whole-blood samples from contacts and suspected cases.

Monkeypox case definitions for the CAR national surveillance program follow international recommendations adapted from WHO, the US Centers for Disease Control and Prevention (CDC), and Nigeria Centre for Disease Control (NCDC; https://ncdc.gov.ng/diseases/info/M). Thus, we considered confirmed case-patients as persons with a history of fever and maculopapular rash on palms and soles and virologic confirmation of MPXV via PCR. We considered suspected cases as illness in persons with clinical manifestations but no virologic confirmation, and we considered contacts to be persons without skin lesions <3 weeks after exposure to a case-patient. We defined the index case as the first human case identified in a village, which might or might not be the primary human case (i.e., the initial case presumed to be from an animal source) (*25*,*26*). Because initial interhuman transmission between the primary and secondary cases might not have been recognized, the index case might not always be the primary case. 

We assessed lesion severity by adding the total number of lesions and scars, then classified severity as mild (<25 lesions), moderate (26–100 lesions), severe (101–250 lesions), or serious (>250 lesions) (*25*). In the absence of easily identifiable scars to determine smallpox vaccination status, we considered persons born before 1980 to have been vaccinated. We defined outbreaks of monkeypox on the basis of >1 confirmed case of human monkeypox; we defined outbreaks of chickenpox on the basis of >1 biologically confirmed chickenpox case. All case-patients received symptomatic and supportive care in accordance with international guidelines for the management of monkeypox disease and were isolated in the hospital for >14 days after virologic diagnosis; isolation was longer when PCR results remained positive. Suspected case-patients were isolated in the nearest healthcare center until they received results of diagnostic procedures. Contacts were quarantined at home for 21 days and received a daily visit from the epidemiologic surveillance point of contact. Smallpox vaccine is not available in CAR for postexposure prophylaxis.

### Laboratory Procedures

WHO recommends PCR of swab samples from pus or crusts for laboratory confirmation of MPXV (https://www.who.int/news-room/fact-sheets/detail/monkeypox). Samples, also are tested for MPXV via intracranial inoculation of suckling mice (*23*). National monkeypox surveillance in CAR uses a quantitative conventional PCR that targets the hemagglutinin gene and part of the A-type inclusion body gene by using generic and clade I primers (*27*), as described in previous outbreak investigations (*18*,*21*,*22*). Case-patients and contacts are also tested for IgG against monkeypox in blood by using an in-house ELISA and antigens from a local MPXV strain (GenBank access no. MN702450) obtained during a previous epidemic (*18**–**22*). This serologic assay has not been formally validated, and cross-reactivity between orthopoxviruses is likely, as reported for other similar assays (*28*).

### Statistical Analysis

We compared groups by using Mann-Whitney or Kruskal-Wallis tests for continuous data or by using χ^2^ test for discrete data. We investigated the monthly number of confirmed monkeypox outbreaks by assuming a Poisson distribution. We used univariable and multivariable logistic regression analyses to investigate factors associated with IgG against MPXV. We performed all statistical analyses in Stata version 15.0 (StataCorp LLC, https://www.stata.com).

### Ethics Considerations

Outbreak investigations were conducted within the framework of the CAR national surveillance program. We obtained authorization to use these data for research purposes from the institutional review board of Institut Pasteur Paris (authorization no. IRB00006966) on January 10, 2020, and from the Comité Ethique et Scientifique of the Université de Bangui on February 21, 2021.

## Results

**Figure 1:**
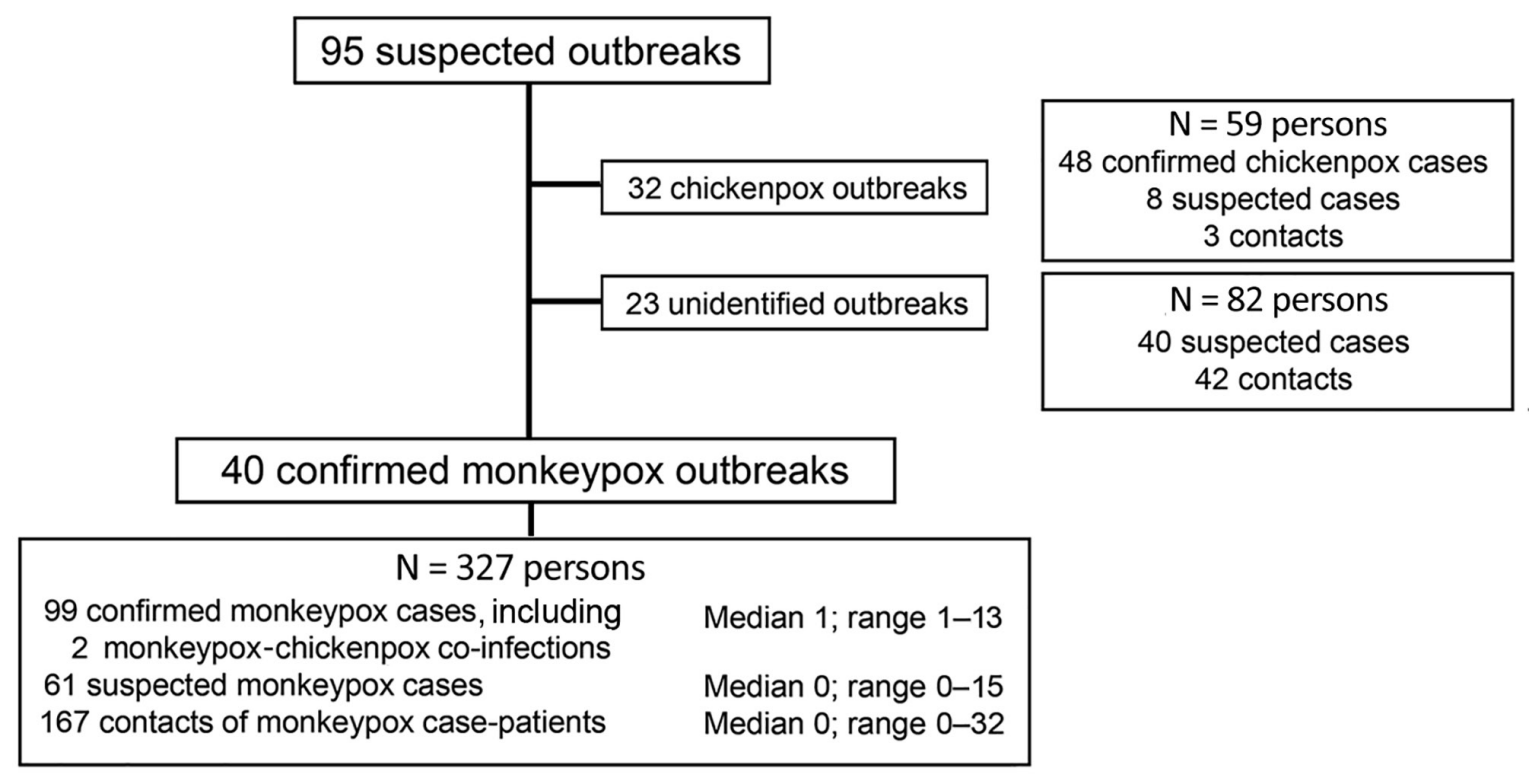
Cases detected and investigated during national monkeypox surveillance, Central African Republic, 2001–2021.

During 2001–2021 the national surveillance system identified 95 suspected monkeypox outbreaks and investigated 468 persons. Of those persons, 99 were confirmed as monkeypox cases, 48 were confirmed as chickenpox cases, 109 were suspected co-infections, and 212 were contacts (Figure 1). From these findings, we identified 40 confirmed monkeypox outbreaks, including 2 persons with monkeypox–chickenpox co-infection, 32 exclusive confirmed chickenpox outbreaks, and 23 outbreaks of undetermined origin without confirmation of monkeypox or chickenpox (Figure 1).

The 40 confirmed monkeypox outbreaks encompassed 327 persons, including 99 confirmed monkeypox cases (including the 2 persons with chickenpox–monkeypox co-infection), 61 suspected monkeypox cases, and 167 contacts (Figure 1; Appendix Table 1). The size of the confirmed monkeypox outbreaks ranged from 1–13 confirmed cases, but no outbreak involved >25 confirmed and suspected cases. We detected 3 chickenpox outbreaks that were spatially and temporally concomitant with 3 confirmed monkeypox outbreaks. The maximum number of contacts investigated in a single confirmed monkeypox outbreak was 32 (Appendix Table 1).

**Figure 2:**
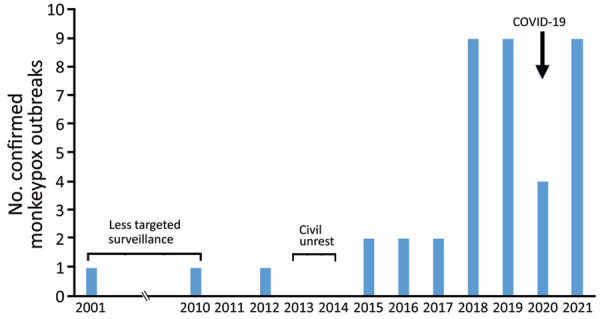
Confirmed outbreaks detected during national monkeypox surveillance, Central African Republic, 2001–2021.

**Figure 3:**
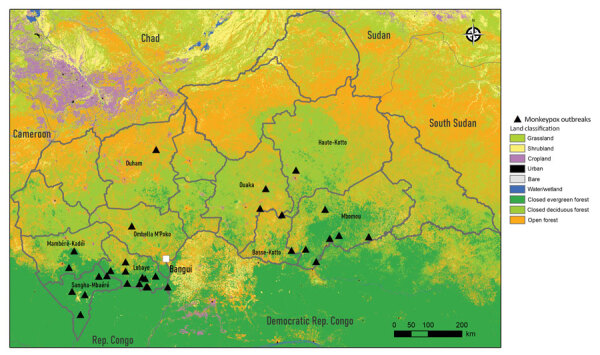
Confirmed outbreaks detected during national monkeypox surveillance, Central African Republic, 2001–2021. Source: Copernicus 2019 Global 100 m Landcover (https://doi.org/10.3390/rs12061044). Rep., Republic.

During 2001–2017, very few (0–2 annually) monkeypox outbreaks were reported. Since 2018, the annual number of outbreaks reported climbed to 9, but a transient decrease occurred in 2020 during the COVID-19 pandemic (Figure 2; Appendix Table 1). Lobaye (40% of outbreaks) and Mbomou (25% of outbreaks), both part of the Congo Basin Forest (Figure 3), were the geographic areas principally affected. Outbreaks mostly occurred in remote rural areas, although a few recent outbreaks occurred in small towns, such as Ippy (population 17,000), Raffaï (population 13,000), and Bania (population 5,000), and in 2 affected artisanal gold-mining areas (Appendix Table 1). Monthly rates of confirmed monkeypox outbreaks were heterogeneous (p = 0.002), and most outbreaks occurred in September (Appendix Figure 1). For 16 outbreaks, index cases described exposure to wildlife, consistent with a zoonotic source (Appendix Table 1).

Among confirmed case-patients, 51 (53.1%) were female (Table; Appendix Figure 2); half were children <16 (interquartile range [IQR] 5.5–28) years of age, 54.4% of whom were male. The 40 outbreak index cases were evenly distributed between the sexes: 19 were male, 19 female, and 2 had missing data for sex; we observed no predominance of a particular age group. Only 3 confirmed case-patients were >42 years of age and we presumed they were vaccinated against smallpox (Table).

**Table:** Characteristics of 327 cases investigated during monkeypox national monkeypox surveillance, Central African Republic, 2001–2021*

Characteristics	Confirmed cases, n = 99	Suspected cases, n = 61	No. contacts, n = 167	p value
Sex				0.6
F	51 (53.1)	37 (60.7)	90 (53.9)	
M	45 (46.9)	24 (39.3)	77 (46.1)	
Data missing	3	0	0	
Median age, y (IQR)	15.5 (5.5–27)	8 (2–23)	27 (14–40)	<0.001
Age group, y				<0.001
0–9	33 (35.9)	30 (50.9)	23 (17)	
10–19	17 (18.5)	9 (15.2)	18 (13.3)	
20–29	22 (23.9)	11 (18.6)	30 (22.2)	
>30	20 (21.7)	9 (15.3)	64 (47.4)	
Missing data	7	2	32	
Born before 1980†				0.001
Y	3 (3.3)	4 (6.8)	28 (20.7)	
N	89 (96.7)	55 (93.2)	107 (79.3)	
Missing data	7	2	32	
Status/occupation				0.001
Child	37 (51.9)	26 (61.9)	5 (13.2)	
Farmer	16 (22.2)	11 (28.2)	15 (39.5)	
Hunter/fisherman	6 (8.3)	0	1 (2.6)	
Healthcare worker	0	1 (2.4)	4 (10.5)	
Mine worker	2 (2.8)	0	6 (15.8)	
Market trader	2 (2.8)	2 (4.8)	0	
Other	9 (12.5)	2 (4.7)	7 (18.4)	
Missing data	27	19	129	
Reported contact with a human case				0.004
Y	44 (65.7)	28 (75.7)	36 (94.7)	
N	23 (34.3)	9 (24.3)	2 (5.3)	
Missing data	32	24	129	
Contact setting				<0.001
Home	40 (95.2)	22 (81.5)	17 (56.7)	
Elsewhere	2 (4.8)	5 (18.5)	13 (43.3)	
Missing data	57	34	137	
Fever before rash				0.1
Y	51 (85)	29 (96.7)	NA	
N	9 (15)	1 (3.3)	NA	
Missing data	39	31	NA	
Diagnostic sample collected				<0.001
Blood	45 (49.5)	53 (86.9)	46 (97.9)	
Pus	32 (35.2)	5 (8.2)	0	
Crust	14 (15.4)	3 (4.9)	1 (2.1)	
Missing data	8	0	120	

*Values indicate no. (%) of available data except as indicated. Number of cases missing data are indicated for each characteristic. NA, not applicable †Presumably persons born before 1980 were vaccinated against smallpox.

**Figure 4:**
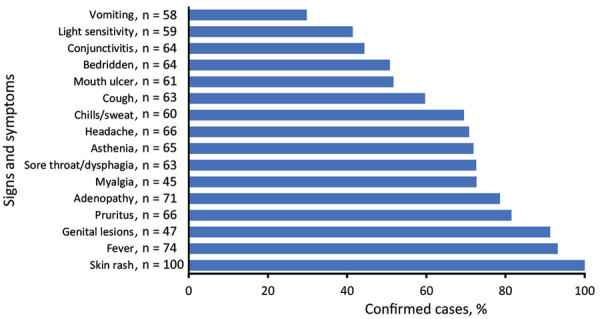
Frequency of signs and symptoms among 99 confirmed monkeypox cases detected during national surveillance, Central African Republic, 2001–2021.

**Figure 5:**
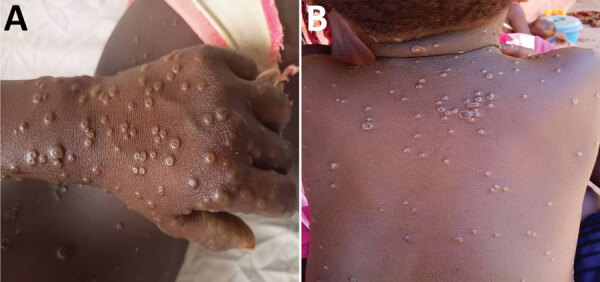
Examples of rash severity detected during national monkeypox surveillance, Central African Republic, 2001–2021. A) Serious rash on a patient’s left hand. Serious rash was reported in 5.7% of cases. B) Moderate rash on a patient’s back. Moderate rash was reported in 57.1% of cases.

**Figure 6:**
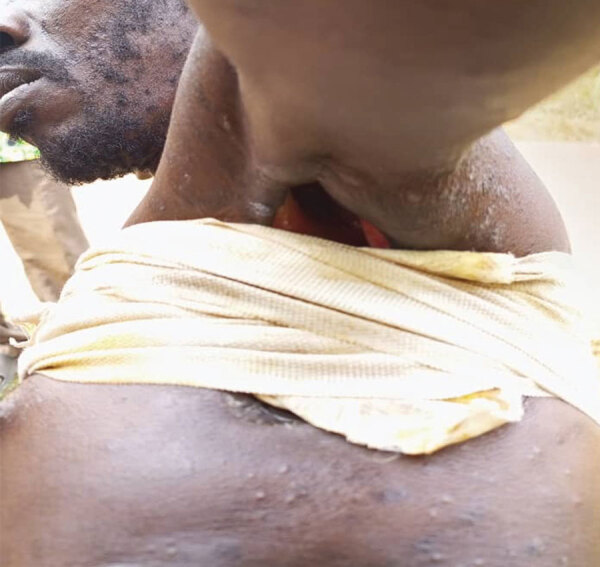
Example of fistulation of axillary adenopathy detected during national monkeypox surveillance, Central African Republic, 2001–2021.

Information about the incubation period, the interval between exposure and symptom onset, was available for 29 persons. The median incubation period was 7 (range 0–17; IQR 1–13) days. The median time from rash onset to sample collection was 9.5 (range 0–31; IQR 5–17) days, and monkeypox was diagnosed via samples of blood in 45 cases, pus in 32 cases, and crusts in 14 cases. All (100%) confirmed case-patients had a rash, and most reported fever (93.2%), pruritus (81.5%), and lymphadenopathy (78.6%) (Figure 4). All case-patients reported a disseminated rash, but 91.3% (42/46) of those for whom information was available had recorded genital lesions. Rashes were graded as moderate (57.1%), severe (34.3%), or serious (5.7%) (Figure 5), and we noted no association between rash intensity and age or sex. Disease severity was not associated with presumed zoonotic or interhuman transmission. Among 18 confirmed case-patients for whom information was available, 11 experienced >1 disease complications, such as septicemia (n = 3), bronchopneumonia (n = 4), dehydration (n = 6), corneal ulceration (n = 2), cutaneous bacterial superinfection (n = 3), fistulation of axillary adenopathy (n = 1) (Figure 6), and keloid healing (n = 4). HIV testing is not systematically performed for monkeypox cases in CAR, and only 1 case of HIV co-infection was identified in a patient who recovered from monkeypox after experiencing a serious rash (>250 lesions). Eight case-patients experienced concomitant malaria.

Among confirmed and suspected monkeypox cases, 12 persons died, 8 children and 4 adults (Table; Appendix Table 2), corresponding to an overall case-fatality ratio (CFR) of 7.5% (12/160) and a CFR of 9.6% (8/83) in children <16 years of age. The CFR was 7.1% (7/99) for confirmed cases and 8.2% (5/61) for suspected cases. Information about the cause of death was available for only 3 (25%) deaths: 1 resulted from a severe cutaneous monkeypox form and septicemia, another from a pulmonary edema, and the third from probable neurologic impairment in a child (*19*) (Appendix Table 2).

Among 327 persons investigated during the 40 confirmed monkeypox outbreaks, serologic results were available for 288 (79 confirmed cases, 48 suspected cases, and 161 contacts); missing blood samples were the main reason serologic results were not available. Of 288 cases with serologic results, 159 (55.2%) tested positive for IgG against monkeypox virus. The median interval between outbreak onset and initial sampling was 15 (IQR 6–28.5) days for the confirmed cases and 39 (IQR 20–46.5) days for the suspected cases or contacts. In a multivariable model adjusted for age, sex, and time from outbreak onset to date of first blood sample collection, monkeypox virus IgG was more frequently detected for confirmed cases than for suspected cases and contacts (odds ratio [OR] 2.04, 95% CI 1.00–4.16) (Appendix Table 3). MPXV IgG positivity was bimodally distributed by age, with peaks at 15–19 years and >45 years of age (Appendix Table 3).

## Discussion

CAR has conducted national monkeypox disease surveillance since 2001 although, surveillance programs initially focused more on other eruptive fevers, such as measles and rubella (*9*). Monkeypox surveillance has been more systematic since 2010, albeit with interruptions during periods of civil unrest (2013–2014). The number of confirmed monkeypox outbreaks reported has increased since 2018, although we noted a transient decline in 2020, probably resulting from disruption of normal activities caused by the COVID-19 pandemic (*29*) and civil unrest surrounding CAR presidential elections in December 2020. Before 2018, no outbreaks had been reported between the outbreak in Sangha Mbaéré prefecture during 1983–1984 and the outbreak in Mbomou prefecture in 2001 (*17*,*18*). We observed a similar pattern in areas of sporadic endemicity, where recent outbreaks occurred in Cameroon in 2018 after 29 years of absence (*30*), in Sierra Leone in 2014 after 44 years (*31*), and in Nigeria in 2017 after 39 years (*32*). More generally, the recent increase in the number of outbreaks in CAR has been mirrored elsewhere, in West Africa and the Congo Basin region, and seems to reflect improvements in surveillance, as well as increased viral circulation in a region experiencing major ecologic disturbances (*33*). Indeed, considerable deforestation and land-use changes have occurred in tropical rainforests in recent decades, causing habitat loss for wildlife and proliferation of several opportunistic species, such as rodents, thus increasing interactions between humans and animals and the risk for zoonotic disease emergence (*34*,*35*).

Since 2001, most confirmed monkeypox outbreaks in CAR have occurred in the Lobaye and Mbomou prefectures, forested regions located at the edge of the Congo Basin Forest, a favorable ecosystem for the suspected animal hosts. These 2 regions border DRC and Congo, and multiple commercial and social exchanges occur between these countries, potentially facilitating viral circulation, as demonstrated in previous phylogenetic studies (*18*). The occurrence of monkeypox predominantly in forested areas is characteristic of outbreaks in Central Africa (*2*) but differs from the distribution observed in Nigeria, where outbreaks have recently shifted to savanna and urban areas (*36*). The distribution shift in Nigeria reveals a change in the epidemiologic features of the disease (*32*,*35*) and an increase in the potential for international spread. In CAR, predominance of monkeypox outbreaks in September, at the end of the rainy season, might reflect the movement of local populations into forested areas for caterpillar picking. During that time of the year, entire families, including children on their school holidays, participate in these activities, which bring them into closer contact with wildlife and the deep forest environment, which could increase the risks for zoonotic and interhuman transmission.

As in DRC (*2*), identifying a zoonotic source for outbreaks in CAR has been difficult; only 16 of the 40 confirmed monkeypox outbreaks we identified included a suspected source. A study of 837 monkeypox cases in Tshuapa Province in DRC revealed a similar pattern: only 36.9% of case-patients reported prior contact with animals, and 33.3% reported contact with a symptomatic human case (*37*). The DRC national surveillance program has revealed multiple wildlife exposures in the populations of forested areas (*13*,*25*). Such exposures might be underreported in CAR, leading to an overestimation of the importance of secondary transmission within households (*38*). However, outbreaks in Central Africa seem to be related to iterative independent spillover events, whereas interhuman transmission seems more likely in the urban context of the disease in Nigeria (*36*). More precise identification of infection sources is essential for guiding specific prevention measures. The 2019 description of a case of monkeypox disease relapse in a UK patient also suggests alternative mechanisms underlying the repetition of outbreaks at the same location (*16*), such as interhuman transmission resuming after a virologic or clinical relapse in patients previously affected, as described for Ebola virus disease (*39*).

Among the 95 suspected monkeypox outbreaks we investigated (Figure 1), the preponderance of outbreaks of undetermined origin could partly be explained by the reliance on blood samples for diagnosis; monkeypox diagnosis on the basis of blood samples is known to be less sensitive than diagnosis through pus or crust samples (*6*). Indeed, for 68.2% (15/22) of monkeypox outbreaks of unknown origin, only blood samples were collected, and no pus or crust samples were tested. This lack of testing might be related to healthcare workers in remote areas who lack knowledge of the type of sampling needed for monkeypox diagnosis and their lack of training and medical materials for pus or crust sampling. Chickenpox and monkeypox outbreaks already were reported to simultaneously occur in villages (*40*–*42*), and co-infection was reported for 1 person, but whether these findings correspond to a true cocirculation of the 2 viruses or to false-positive results for chickenpox or monkeypox remains unclear. Concomitant malaria was also reported, and a similar case has been reported in DRC (P.R. Pittman et al., unpub. data, https://doi.org/10.1101/2022.05.26.22273379).

The age and sex distribution of confirmed case-patients might reflect the nature of exposure because young boys traditionally are more likely to have contact with infected animals through playing and hunting (*13*,*43*), whereas women more likely to be exposed through caring for ill persons (*44*). These 2 groups have been shown to be predominantly affected in Central Africa (*8*,*26*,*40*); however, outbreaks in West Africa have shown different patterns, in which the age distribution is older (median age 29 years [*1*]) and most (69%) cases occur among male persons (*32*).

The overall clinical description of cases in the CAR national surveillance program is consistent with reports for other endemic countries, except for the frequency of genital lesions, which were common among patients who were asked in CAR but were more rarely (5%–68%) reported elsewhere (*25*,*45*). Several authors have already suggested that skin-to-skin contact or contact with genital secretions during sexual intercourse might have played a role in transmission in the monkeypox outbreak in Nigeria (*46*). Thus, endemic settings might need improved documentation of genital lesions. The recent monkeypox outbreak in countries outside sub-Saharan Africa, characterized by isolated genital lesions and a predominance of cases in MSM, supports the hypothesis of transmission through close and intimate contact during sexual intercourse (*1*).

The CFR (7.5%) detected in CAR was toward the upper end of the range, as expected for clade I (*3*), and was high (9.6%) for children, as reported elsewhere (*34*,*47*). This CFR is greater than that reported for epidemics in Nigeria (2.8% and 6% during 2017–2018) (*32*,*48*) and greater than CFRs reported since May 2022 from countries outside Africa (*12*); by September 17, 2022, CDC reported only 9 monkeypox deaths among >60,703 diagnosed cases in nonendemic countries (https://www.cdc.gov/poxvirus/monkeypox/response/2022/world-map.html). In endemic settings, CFR has been difficult to accurately estimate (*2*), and some deaths might have occurred before investigations began for those outbreaks, resulting in an underestimation of the number of cases. Also, stratifying CFR by HIV status would have been useful. Unfortunately, reluctance for testing among the local population in CAR limits available information about HIV status.

Smallpox vaccination appears likely to have a protective effect against monkeypox, particularly because we noted only 3 cases among persons born before 1980, the year in which vaccination campaigns with live-attenuated vaccinia virus ended in CAR. Thus, the population at risk for *Orthopoxvirus* infection would be expected to increase over time because fewer persons will be protected by vaccination. Over half the national surveillance population in our study tested positive for IgG against MPXV, as reported in other investigations of serologic response to orthopoxviruses in endemic countries from West or Central Africa (*49*,*50*), particularly in forested areas (*35*). However, in a context of serologic cross-reactions between *Orthopoxvirus* infections (*28*), the antibody peak we observed among persons 15–19 years of age might correspond to childhood exposure to monkeypox or other orthopoxviruses, and the peak among persons >45 years of age might correspond to smallpox vaccination.

In conclusion, characterization of the epidemiologic features of monkeypox in Africa and analysis of the ongoing outbreak outside sub-Saharan Africa is essential. Indeed, the recent increase in monkeypox virus circulation in CAR should be carefully considered in the context of decreasing cross-protective immunity from smallpox vaccination after 1980, the increased deforestation and land use changes in tropical forest, and the potential local and international effects associated with high lethality of clade I. Further investigation of zoonotic sources of infection and the environmental changes involved could enable design of appropriate preventive measures for avoiding expansion of this threatening clade into local communities and beyond endemic areas.

Appendix Additional information on national monkeypox surveillance, Central African Republic, 2001–2021.
