# Supplementary material for: National Monkeypox Surveillance, Central African Republic, 2001–2021
Source: Emerg Infect Dis. 2022 Dec;28(12):2435–45. doi: 10.3201/eid2812.220897 (PMC9707566; doi:10.3201/eid2812.220897)
Supplement: Appendix — Additional information on national monkeypox surveillance, Central African Republic, 2001–2021. [file 22-0897-Techapp-s1.pdf]

Article DOI: https://doi.org/10.3201/eid2812.220897

National Monkeypox Surveillance, Central African Republic, 2001–2021

Appendix

Appendix Table 1. Description of the 40 confirmed outbreaks identified through national monkeypox surveillance, Central African Republic, 2001–2021*

Date	Village (ref. no.)	Prefecture	Cases		Contacts	Total	Deaths	Zoonotic transmission	Interhuman transmission	Environment
			Conf.	Susp.						
2001 Aug	Bangassou (*1*,*2*,*3*)	Mbomou	3	0	0	3	2	Dead NHP	Intrafamily	Forest
2010 Jun	Mongoumba (*2*,*3*)	Lobaye	1	0	0	1	0	*Cephalophus*†	NK	Forest
2012 Apr	Batangafo (*2*)	Ouham	2	0	0	2	0	Squirrel bite	NK	Savanna
2015 Dec	Bria (*3*,*4*)	Haute Kotto	1	0	0	1	1	*Thryonomys*‡	Intrafamily	Not specified
2015 Dec	Madigui (*3*,*4*,*5*)	Mbomou	3	3	0	6	3	*Thryonomys*‡	Intrafamily/nosocomial	Not specified
2016 Jan	Bandoufou (*5*)	Mbomou	1	0	0	1	0	NHP	NK	Not specified
2016 Aug	Alindao (*4*,*6*)	Basse Kotto	3	7	0	10	2	NHP, dead *Xerus erythropus*	Patient’s burial	Forest
2017 Jan	Bao (*7*)	Mbomou	5	0	0	5	0	NK	NK	Not specified
2017 Apr	Machado	Lobaye	1	1	8	10	0	NK	Intrafamily	Forest
2018 Feb	Ippy/Raffaï (*3*,*7*)	Mbomou	9	2	7	18	0	Fish	Intrafamily	Urban
2018 Jun	Bokoka (*3*)	Lobaye	3	3	5	11	0	NK	Intrafamily	Forest
2018 Aug	Raffaï	Mbomou	1	0	0	1	0	NK	NK	Urban
2018 Sep	Bangassou	Mbomou	1	0	0	1	0	NK	NK	Not specified
2018 Sep	Bakouma	Mbomou	1	0	0	1	0	NK	NK	Not specified
2018 Sep	Zomea (*8*)	Lobaye	6	0	32	38	0	*Civettictis civetta*, *Cricetomys emini*, *Funisciurus anerythrus*	Intrafamily	Forest, sawmill
2018 Oct	Ouenzengue	Lobaye	1	0	4	5	0	NK	NK	Forest
2018 Oct	Bangassou	Mbomou	1	0	0	1	0	NK	NK	Not specified
2018 Nov	Bossembele	Ombella M’Poko	5	0	15	20	0	NK	Intrafamily	Not specified
2019 Jan	Ouadjimi	Ouaka	1	3	0	4	0	NK	Intrafamily	Not specified
2019 Feb	Boda	Lobaye	1	15	10	26	0	NK	NK	Forest
2019 Aug	Bomango	Lobaye	1	0	0	1	0	Rodent	NK	Forest
2019 Sep	Loko	Lobaye	4	0	24	28	1	NK	Intrafamily	Forest

Date	Village (ref. no.)	Prefecture	Cases		Contacts	Total	Deaths	Zoonotic transmission	Interhuman transmission	Environment
			Conf.	Susp.						
2019 Sep	Gboloko	Lobaye	1	0	0	1	1	NK	NK	Forest
2019 Sep	Ndolobo	Lobaye	2	0	5	7	0	Rodent, NHP	Intrafamily	Forest
2019 Sep	Moloukou	Lobaye	1	0	9	10	0	NK	NK	Forest
2019 Sep	M'Baïki	Lobaye	1	0	0	1	0	NK	NK	Forest
2019 Oct	Moboma	Lobaye	3	0	0	3	0	NK	NK	Forest
2020 Nov	Mokongo	Lobaye	2	0	0	2	0	Rodent	NK	Forest
2020 Nov	Bambio	Sangha Mbaéré	2	0	0	2	0	NK	NK	Forest, sawmill
2020 Nov	Nola	Sangha Mbaéré	1	2	0	3	0	NK	NK	Forest
2020 Dec	Kembe	Mbomou	3	0	0	3	0	NK	NK	Not specified
2021 Feb	Bagawa	Haute Kotto	1	2	0	3	0	NK	NK	Not specified
2021 Aug	Gbodo	Mbomou	3	0	1	4	0	NK	NK	Not specified
2021 Sep	Massangaï	Sangha Mbaéré	4	3	2	9	0	NK	NK	Forest
2021 Sep	Moloukou	Lobaye	2	4	21	27	1	NK	Intrafamily	Forest
2021 Sep	SCED Ndelengue	Sangha Mbaéré	1	0	1	2	0	NK	NK	Sawmill
2021 Oct	Grima	Lobaye	2	2	7	11	0	Dead NHP, dead palm rat	Intrafamily	Forest
2021 Nov	Bania	Mambéré Kadéi	13	12	17	42	1	NHP	Intrafamily	Savanah
2021 Nov	Nazembe	Mambéré Kadéi	1	2	0	3	0	Fish	NK	Gold mining
2021 Dec	Balego, Bayanga	Mambéré Kadéi	1	0	0	1	0	*Atherurus africanus*	NK	Gold mining

*Conf., confirmed; NHP, non-human primate; NK, not known; Susp., suspected.
†*Cephalophus sylvacultor*
‡*Thryonomys*: aulacode

Appendix Table 2. Characteristics among 12 deaths related to confirmed outbreaks identified through national monkeypox surveillance, Central African Republic, 2001–2021*

Year	Village	Confirmed or suspected case	Age, y/sex	HIV status	Cause of death	Reference no.
2001	Bangassou	Confirmed	8/M	NK	NK	(*1*,*2*,*3*)
2001	Bangassou	Confirmed	9/M	NK	NK	(*1*,*2*,*3*)
2015	Bria	Confirmed	8/M	NK	NK	(*3*,*4*,*5*)
2015	Madigui/Bakouma	Confirmed	27/F	NK	NK	(*3*,*4*,*5*)
2016	Madigui/Bakouma	Suspected	15 mo./M	Negative	Agitation and hypotonia	(*3*,*4*,*5*)
2016	Madigui/Bakouma	Suspected	5./M	Negative	Pulmonary edema and hypothermia	(*3*,*4*,*5*)
2016	Rehou 4/Alindao	Suspected	child/NK	NK	NK	(*3*,6
2016	Rehou 4/Alindao	Suspected	12 mo./NK	NK	NK	(*3*,6
2019	Bossembo/Loko	Suspected	1/M	NK	NK	Database
2019	Gboloko	Confirmed	36/F	NK	NK	Database
2021	Bakota/Moloukou	Confirmed	41/F	NK	Septicemia, severe form	Database
2021	Bania	Confirmed	23/F	NK	NK	Database

*NK, not known.

Appendix Table 3. Risk factors for monkeypox IgG seropositivity among tested persons identified through national monkeypox surveillance, Central African Republic, 2001–2021*

Factors	Adjusted OR (95% CI)
Case status	
Confirmed	2.04 (1.00–4.16)
Suspected and contacts	Referent
Sex	
F	1.33 (0.74–2.40)
M	Referent
Age group, y	
0–4	Referent
5–9	6.0 (1.85–19.48)
10–14	6.36 (1.87–21.66)
15–19	26.51 (5.36–131.28)
20–24	8.83 (2.69–29.02)
25-29	5.31 (1.60–17.64)
30–34	7.33 (2.00–26.37)
35–39	6.21 (1.72–22.42)
40–44	6.64 (1.62–27.15)
45–49	15.46 (2.34−102.3)
>50	9.36 (2.62–33.4)
Time from index case symptom onset and sampling, d	
0–7	Referent
8–44	0.73 (0.21–2.54)
5–21	0.75 (0.27–2.08)
2–28	1.80 (0.57–5.69)
29–35	0.91 (2.19–25.18)
36–42	7.43 (2.19–25.18)
43–49	1.77 (0.66–4.73)
50–56	0.76 (0.17–3.37)
>57	1.33 (0.36–4.98)

*Values calculated through multivariable analysis. OR, odds ratio.

References

1. Durski KN, McCollum AM, Nakazawa Y, Petersen BW, Reynolds MG, Briand S, et al. Emergence of monkeypox—West and Central Africa, 1970–2017. MMWR Morb Mortal Wkly Rep. 2018;67:306–10. PubMed https://doi.org/10.15585/mmwr.mm6710a5

2. Berthet N, Nakouné E, Whist E, Selekon B, Burguière AM, Manuguerra JC, et al. Maculopapular lesions in the Central African Republic. Lancet. 2011;378:1354. PubMed https://doi.org/10.1016/S0140-6736(11)61142-2

3. Beer EM, Rao VB. A systematic review of the epidemiology of human monkeypox outbreaks and implications for outbreak strategy. PLoS Negl Trop Dis. 2019;13:e0007791. PubMed https://doi.org/10.1371/journal.pntd.0007791

4. Nakoune E, Lampaert E, Ndjapou SG, Janssens C, Zuniga I, Van Herp M, et al. A Nosocomial Outbreak of Human Monkeypox in the Central African Republic. Open Forum Infect Dis. 2017;4:ofx168. PubMed https://doi.org/10.1093/ofid/ofx168

5. Kalthan E, Dondo-Fongbia JP, Yambele S, Dieu-Creer LR, Zepio R, Pamatika CM. Twelve cases of monkeypox virus outbreak in Bangassou District (Central African Republic) in December 2015 [in French]. Bull Soc Pathol Exot. 2016;109:358–63. PubMed https://doi.org/10.1007/s13149-016-0516-z

6. Kalthan E, Tenguere J, Ndjapou SG, Koyazengbe TA, Mbomba J, Marada RM, et al. Investigation of an outbreak of monkeypox in an area occupied by armed groups, Central African Republic. Med Mal Infect. 2018;48:263–8. PubMed https://doi.org/10.1016/j.medmal.2018.02.010

7. Berthet N, Descorps-Declère S, Besombes C, Curaudeau M, Nkili Meyong AA, Selekon B, et al. Genomic history of human monkey pox infections in the Central African Republic between 2001 and 2018. Sci Rep. 2021;11:13085. PubMed https://doi.org/10.1038/s41598-021-92315-8

8. Besombes C, Gonofio E, Konamna X, Selekon B, Gessain A, Berthet N, et al. Intrafamily transmission of monkeypox virus, Central African Republic, 2018. Emerg Infect Dis. 2019;25:1602–4. PubMed https://doi.org/10.3201/eid2508.190112

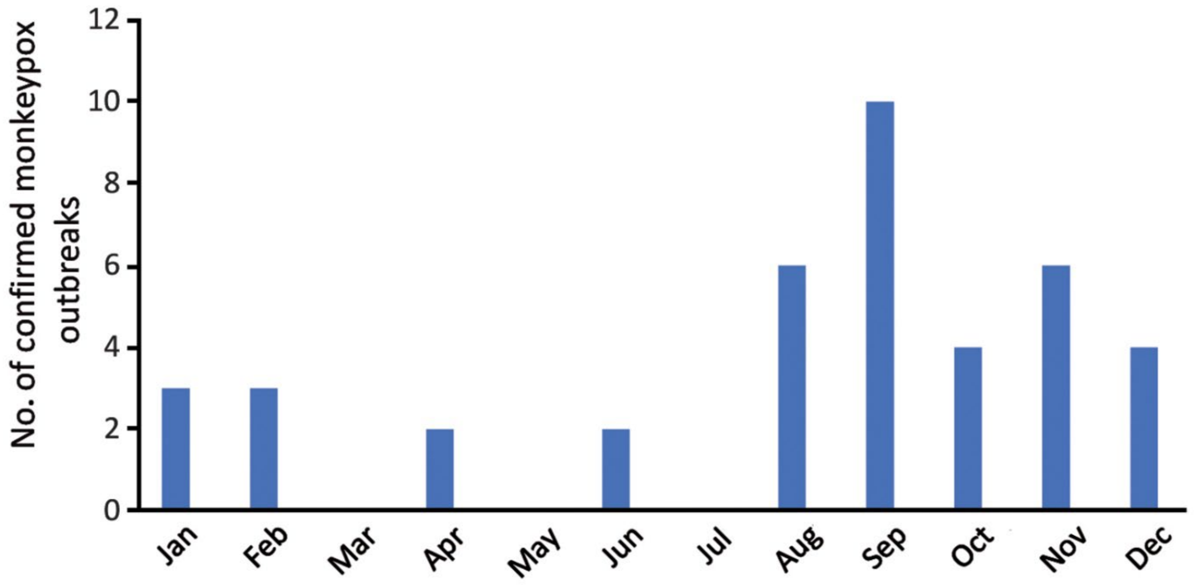

Appendix Figure 1. Distribution of months in which outbreaks occurred during national monkeypox surveillance, Central African Republic, 2001–2021.

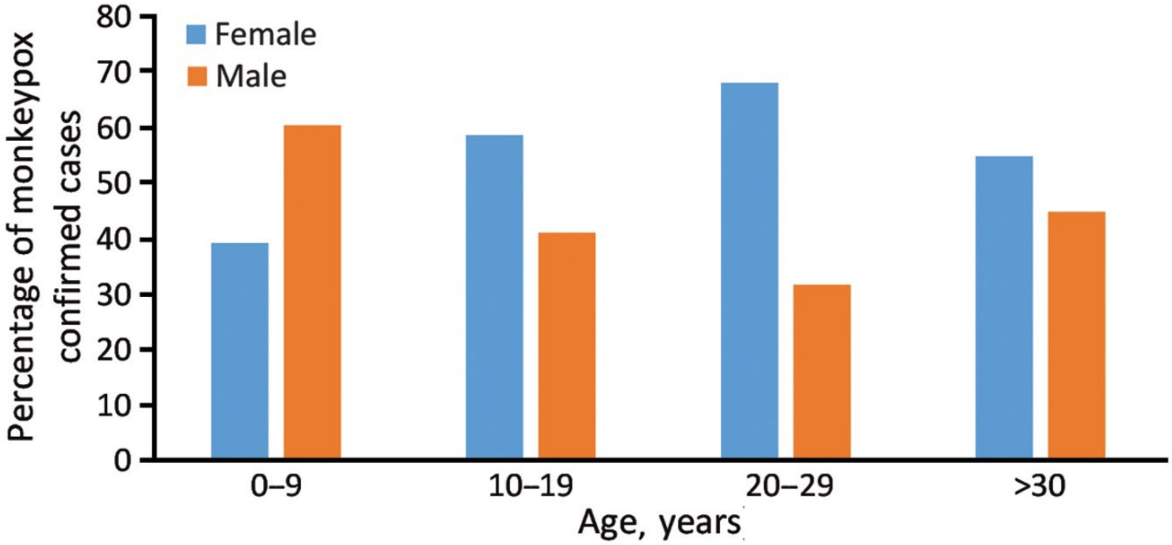

Appendix Figure 2. Age and sex of 99 cases detected and investigated during national monkeypox surveillance, Central African Republic, 2001–2021.
